# Medical Management of Pyometra in the Delayed Postoperative Period

**DOI:** 10.1155/2021/7995348

**Published:** 2021-10-28

**Authors:** Natalie A. Vukmer, Heather Urrego, A. Mitch Dizon

**Affiliations:** Department of Obstetrics & Gynecology, University of Tennessee College of Medicine, Chattanooga, TN, USA

## Abstract

Pyometra is a rare condition in which purulent material becomes entrapped within the uterine cavity. If unrecognized in a timely fashion, life-threatening complications can arise. The following is a case report of a 50-year-old female who presented to the emergency department with abdominal pain and heavy vaginal bleeding. She was diagnosed with a pyometra based on imaging and treated conservatively with antibiotics. The patient ultimately had an uncomplicated hysterectomy with resolution of normal female pelvic anatomy prior to surgery. Pyometra should be considered when women present with diffuse abdominal pain or peritonitis. As demonstrated in this report, early detection and conservative management may help prevent serious complications such as uterine perforation, lead to shorter hospital stays, and result in safer operative management.

## 1. Introduction

Pyometra is a gynecologic-specific condition where an infection develops within the uterus, and natural drainage is disrupted. Although this phenomenon is well documented in female dogs, the infection is rare in humans with a reported incidence of 0.1-0.2% [1]. If unrecognized, pyometra can lead to uterine perforation and is associated with a poor prognosis. A review conducted in 2012 reported only 81 cases of uterine rupture between 1949 and 2011 and a 25% mortality rate [2].

Most cases of pyometra occur in elderly, postmenopausal women with recent intrauterine surgery or women with comorbidities such as impaired glucose tolerance, hypertension, rheumatoid arthritis, or osteoarthritis [2]. Patients often present with abdominal pain, fever, an enlarged uterus, or signs of pelvic infection such as foul-smelling vaginal discharge [3]. However, many of the presenting symptoms may be vague and nonspecific. When suspicion for pyometra occurs, an ultrasound or CT scan is the imaging modalities to best detect this infection.

Common associations of pyometra are benign tumors, cervical occlusion after surgery, and history of pelvic radiation or malignant diseases [4]. Rare cases have demonstrated these infections can progress and ultimately lead to a ruptured uterus, emergent surgery, and possibly death [2, 5]. Therefore, early detection and appropriate management of this condition are imperative.

## 2. Case

A 50-year-old postmenopausal woman presented to our outpatient clinic with postmenopausal bleeding. She had a past medical history of hypertension, diabetes mellitus with a hemoglobin A1c of 6.8, hyperlipidemia, temporomandibular arthritis, carpal tunnel syndrome, and transient ischemic attack following mitral valve replacement requiring Coumadin therapy. Her surgical history was significant for a mitral valve replacement and bilateral tubal ligation.

**Figure 1:**
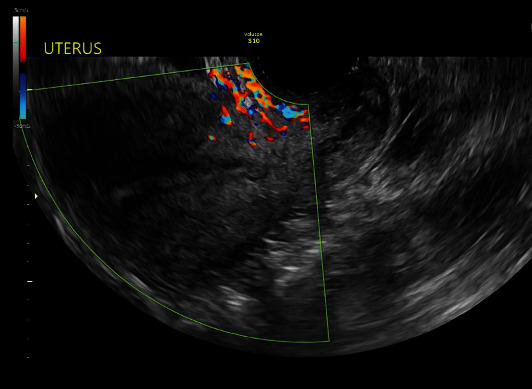
Transvaginal ultrasound performed in clinic for evaluation of abnormal uterine bleeding. Endometrial thickness was noted to be 4.70 mm with a uterine length of 9.1 cm and width of 8.2 cm.

The initial workup of her postmenopausal bleeding included a pelvic ultrasound and endometrial biopsy. The ultrasound revealed a heterogeneous and enlarged uterus with an endometrial stripe of 4.7 mm. The myometrium did not appear hypervascular. No free fluid was noted in the pelvis (Figure 1). The right ovary was difficult to visualize, however, two small simple-appearing cysts were visualized on her left ovary. The endometrial biopsy showed predominantly blood and scant inactive endometrium with extensive glandular and stromal breakdown. Once pathology was confirmed to be benign, the patient was started on medroxyprogesterone to decrease bleeding in preparation for her initial procedure.

The patient underwent an uncomplicated hysteroscopy, dilation and curettage, and endometrial ablation. No cervical stenosis was noted. Evaluation of the endometrial cavity revealed a normal cavity without polyps or other cavity-distorting pathology, and the endometrial lining appeared thin. A hysteroscopy was performed after the ablation which noted a small area at the uterine fundus to be incompletely ablated.

The patient was contacted on postoperative day one and reported she was feeling well with minimal vaginal bleeding. She was instructed to discontinue the medroxyprogesterone and restart her Coumadin. She presented for a routine postoperative visit on day seven and continued to endorse minimal vaginal bleeding. Her PT/INR was monitored by the Coumadin clinic and was stable.

**Figure 2:**
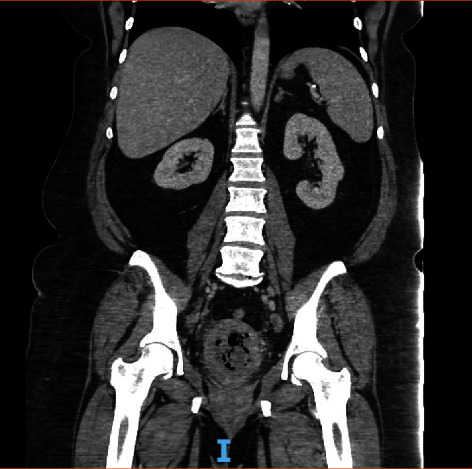
CT scan with contrast at time of presentation to the emergency department. Gas fluid collection was noted to measure up to 13 cm in length, 6 cm in width, and 5 cm in height. Findings concerning for abscess/pyometra.

**Figure 3:**
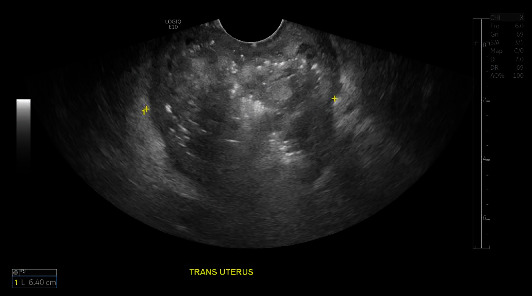
Transvaginal ultrasound performed in the emergency department. Foul-smelling discharge noted in addition to heterogeneous material and air within the uterus.

On postoperative day 29, the patient presented to the emergency department with complaints of pelvic pain, vaginal bleeding, abnormal vaginal discharge, and a fever at home of 100.9 F. A physical exam revealed thick, foul-smelling discharge, and copious dark blood clots. The cervical os was dilated, and there was significant tenderness of pelvic organs on exam. A CT scan of the abdomen and pelvis with contrast (Figure 2) and pelvic ultrasound (Figure 3) was consistent with a pyometra. A cervical culture grew *Streptococcus agalactiae*.

The patient received one dose of vancomycin and piperacillin/tazobactam in the emergency department prior to admission. She was transitioned to intravenous cefoxitin and doxycycline for two days while hospitalized. Her vaginal bleeding and pain improved significantly, and she was discharged home with oral doxycycline and metronidazole to complete a fourteen-day course. She restarted medroxyprogesterone for bleeding control.

**Figure 4:**
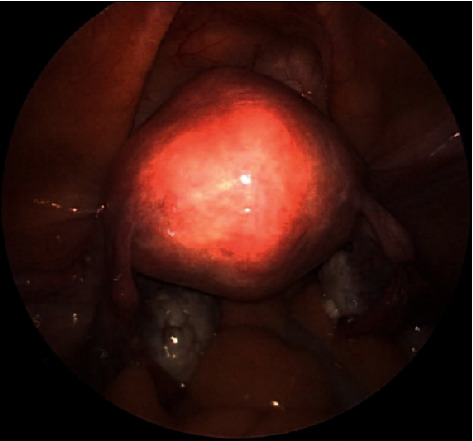
Pelvic anatomy at the time of hysterectomy, bilateral salpingectomy. Minimal adhesions noted and uterus did not appear enlarged.

Despite her improvement, she desired definitive management with a hysterectomy. The total laparoscopic hysterectomy and bilateral salpingectomy was uncomplicated with a normal-appearing uterus and minimal pelvic adhesions (Figure 4). She had an uncomplicated postoperative course. The final pathology demonstrated a degenerated endometrium consistent with previous instrumentation/ablation, otherwise normal cervix, uterus, and fallopian tubes.

## 3. Discussion

This case report details initial conservative management of pyometra that may allow for safer definitive management after recovery. It is possible that this case started as a hematometra that further progressed to a pyometra given this patient's Coumadin use, history of bilateral tubal ligation, and recent endometrial ablation. The hematometra would have provided an excellent medium for bacterial growth. Regardless, we believe that a stable patient with pyometra without a preexisting hematometra could be managed in a similar fashion.

Commonly isolated organisms from pyometras include *Streptococcus* species, *Bacteroides fragilis*, and *Escherichia coli* [1]. The culture in this case was collected during a speculum exam of the products extruding from the cervical os. The culture grew *Streptococcus agalactiae*. She was treated conservatively and her condition improved.

Pyometra is a rare condition that can be life-threatening. Active pelvic infections can increase the risk of bleeding, injury to surrounding organs, and further infections. Given the rarity of this condition, improvements in management protocols are needed.

Surgical management should not be postponed in rare cases of pyometra that present with a surgical abdomen or do not improve with antibiotic therapy. However, if a patient is stable, administration of intravenous antibiotics may be a reasonable choice—especially in poor surgical candidates or women who wish to preserve fertility. Ultimately, these patients may be followed in an outpatient setting to complete antibiotic therapy.

Given the increased risk of pyometra development from cervical stenosis/occlusion, there should be a high suspicion for malignancy in spontaneous presentations [6]. Therefore, close outpatient follow-up and histological review are imperative.

## Data Availability

The data presented in this case report is available from the corresponding author upon reasonable request.
